# Dual-threshold plasma Aβ oligomerization for postoperative delirium risk stratification

**DOI:** 10.1093/ageing/afag106

**Published:** 2026-04-22

**Authors:** YoungSoon Yang, Ki Jin Jung, Yong Tae Kwak

**Affiliations:** Department of Neurology, Soonchunhyang University, Cheonan-si, Chungcheongnam-do 31151, Korea; Department of Orthopedic Surgery, Soonchunhyang University, Cheonan-si, Chungcheongnam-do 31151, Korea; Department of Neurology, Hyoja Geriatric Hospital, 1-30, Jungbu-daero 874beon-gil, Giheung-gu, Yongin-si, Gyeonggi-do 17089, Korea

**Keywords:** postoperative delirium, MDS-OAβ, amyloid-β oligomers, ROC curve, biomarkers, older people

## Abstract

**Background:**

Postoperative delirium is common in older surgical patients, but simple blood tests to identify risk are lacking. Plasma amyloid-β oligomers measured by multimer detection (MDS-OAβ) may reflect neurodegenerative vulnerability.

**Methods:**

We enrolled 101 patients aged ≥65 years undergoing elective orthopaedic surgery with general anaesthesia. Blood was drawn preoperatively and at first delirium diagnosis or on postoperative Day 4 if no delirium. MDS-OAβ was quantified blinded. Delirium was assessed daily on postoperative Days 1–3 (DRS-R-98 and DSM). Propensity-score matching on APOE ε4 status and clinical covariates addressed Alzheimer-type vulnerability. Discrimination and thresholds (0.60, 0.72, 0.85 ng/ml) were evaluated using logistic regression and ROC analyses.

**Results:**

Among 101 patients (44 with delirium; 57 without), preoperative MDS-OAβ concentrations were higher in those who developed delirium and correlated with delirium severity. In the overall cohort, preoperative MDS-OAβ discriminated delirium with an area under the curve of 0.855 (95% CI 0.777–0.919); in a pooled postoperative dataset (*n* = 205), discrimination was similar (AUC 0.884, 95% CI 0.837–0.925). The dual-threshold approach identified a low-risk group with high negative predictive value and a high-risk group with high positive predictive value, leaving an intermediate group for closer observation.

**Conclusions:**

Preoperative plasma MDS-OAβ may provide a scalable biomarker for perioperative risk stratification of postoperative delirium in older adults, supporting a dual-threshold strategy for targeted prevention and monitoring. Low MDS-OAβ values indicate lower risk but do not exclude POD; biomarker-guided stratification should complement, not replace, routine perioperative delirium surveillance.

Key Points

- Postoperative delirium is common in older surgical patients and is poorly predicted by existing clinical risk scores.
- Plasma amyloid-β oligomerization (MDS-OAβ) may indicate underlying neurodegenerative vulnerability to postoperative delirium.
- In this single-centre cohort, higher preoperative MDS-OAβ was associated with postoperative delirium using dual low-risk and high-risk thresholds to guide prevention and monitoring intensity.
- Preoperative MDS-OAβ may offer a scalable blood test for perioperative risk stratification and targeted delirium prevention in older adults.

## Introduction

Postoperative delirium (POD) is among the most frequent and consequential complications in older surgical patients, associated with prolonged length of stay, major in-hospital complications, non-home discharge and excess short-term mortality [1–3]. In a national cohort of >5.5 million non-cardiac surgical admissions, POD was linked to ~3.5-fold higher odds of death or major complications and nearly four-fold higher odds of non-home discharge, underscoring the need for pragmatic, scalable risk-stratification that can be deployed before or immediately after surgery in adults aged ≥65 years [3]. Over the last decade, attempts to connect POD with Alzheimer’s disease (AD) biology have converged on a vulnerability model in which latent neurodegenerative change lowers the threshold for perioperative stressors to precipitate delirium in elders. Prospective data and quantitative synthesis indicate that lower preoperative CSF Aβ42 is negatively associated with incident POD and early postoperative cognitive decline [4, 5]. By contrast, small-scale positron emission tomography (PET) studies have yielded inconsistent associations between cortical amyloidosis and POD severity, and recent plasma work using monomeric Aβ42/40 was negative [6–8]. Taken together—the heterogeneity observed for CSF tau species and other candidate markers, the practical constraints of routine CSF sampling, and the inconsistent reports for blood-based monomeric Aβ42/40—these mixed results underscore the need for a scalable perioperative biomarker. In parallel, neurophysiologic predictors such as perioperative electroencephalography (EEG) show promise but require specialist infrastructure that limits routine adoption [9–11]. The translational gap therefore persists for a simple, blood-based test that can support scalable perioperative risk stratification and guide the intensity of delirium prevention and monitoring.

Within this context, soluble oligomeric amyloid-β (Aβ) is biologically compelling. Across the AD continuum, oligomers—rather than monomers or plaque burden—track more closely with synaptic dysfunction and clinical status. The Multimer Detection System for oligomeric Aβ (MDS-OAβ) quantifies the oligomerization tendency of plasma Aβ and has shown clinical signal across cognitive staging and amyloid burden [12, 13]. ApoE-ε4–related differences in oligomer handling further reinforce biological plausibility for an oligomer-centric read-out in older adults [14]. In Korean clinical practice, MDS-OAβ is already used adjunctively when AD is suspected, with a commonly referenced clinical cut-off of 0.78 ng/ml [15–18]. This external benchmark is informative for perioperative applications, where disease prevalence and action thresholds differ from memory-clinic diagnosis.

Building on this rationale, our programmatic work has evaluated plasma Aβ oligomerization as a perioperative risk marker for POD in older adults. In an initial retrospective orthopaedic-predominant cohort, patients who developed POD had higher MDS-OAβ than those who did not, with graded associations to delirium severity [19]. A subsequent prospective pilot with standardised preoperative and postoperative sampling and blinded adjudication again found that higher plasma MDS-OAβ was associated with incident POD, that pre- and postoperative values were tightly correlated, and that the pre-to-post change was small—features more consistent with a trait-like vulnerability than a transient postsurgical artefact [20].

We therefore designed the present prospective study to test whether preoperative MDS-OAβ can support a dual-threshold risk-stratification strategy for POD: a low threshold calibrated for high sensitivity/negative predictive value (NPV) to identify a low-risk group suitable for standard bundles, and a high threshold calibrated for high specificity/positive predictive value (PPV) to identify a high-risk group for targeted prevention [21, 22]. We emphasise that MDS-OAβ is evaluated here as an adjunct vulnerability marker for POD risk stratification, not as a diagnostic test for AD.

## Methods

### Participants and procedures

Between March and September 2025, we prospectively enrolled consecutive patients aged ≥65 years who underwent general anaesthesia for elective orthopaedic procedures (Table 1) at a single tertiary university-affiliated hospital in South Korea. Given the feasibility design with consecutive enrolment over a fixed period, no *a priori* power calculation was performed; sample size was pragmatic. Patients were approached preoperatively if postoperative delirium assessments during the index admission were feasible. Exclusion criteria were: (i) delirium at baseline, (ii) global cognitive impairment defined as Korean Mini-Mental State Examination (K-MMSE) <24 or known dementia due to any aetiology, (iii) daily use of tranquillisers or antipsychotics and/or heavy alcohol consumption, (iv) known central nervous system diseases (including major psychiatric illness) that could confound delirium evaluation and (v) inability to complete neuropsychological testing (e.g. prolonged postoperative intubation with planned sedation, severe hearing/visual deficits). On the day before surgery, participants completed the K-MMSE and routine preoperative laboratory tests. APOE genotyping was obtained for all participants. The study protocol was approved by the Institutional Review Board and written informed consent was secured from each participant or a legally authorised representative. Given the modest sample size of the present prospective cohort, we performed a sensitivity analysis that pooled postoperative MDS-OAβ measurements from our prior retrospective study [19] with the postoperative measurements obtained in the current cohort, as both datasets were collected under largely identical definitions and procedures.

**Table 1:** Clinical characteristics and MDS-OAβ value in the overall study population, patients with and without postoperative delirium

Variables	Overall, *n* = 101	Delirium		*P*-value^a^
		Yes, *n* = 44	No, *n* = 57	
Age, years	76.3 ± 5.9	76.6 ± 5.8	76.0 ± 6.0	.629
Female gender (%)	46 (45.5%)	17 (38.6%)	29 (50.9%)	.235
Education	8.4 ± 3.2	8.0 ± 2.9	8.8 ± 3.3	.179
MMSE	28.2 ± 1.2	28.0 ± 1.3	28.3 ± 1.1	.233
Number of ApoE4 gene	0.3 ± 0.6	0.4 ± 0.7	0.2 ± 0.5	.140
Surgery name				
THA	56	26	30	–
KAM	12	6	6	–
ARCR	11	4	7	–
TKA	10	4	6	–
CAIO	9	3	6	–
EABM	3	1	2	–
K-DRS-R-98severity	12.7 ± 10.2	24.0 ± 3.2	4.0 ± 1.5	.000
K-DRS-R-98total	14.1 ± 11.6	26.9 ± 3.5	4.2 ± 1.6	.000
preMDS-OAβ(ng/ml)	0.65 ± 0.23	0.80 ± 0.16	0.53 ± 0.20	.000
postMDS-OAβ(ng/ml)	0.65 ± 0.23	0.82 ± 0.16^b^	0.52 ± 0.19	.000
delta MDS-OAβ(ng/ml)	0.01 ± 0.04	0.01 ± 0.04	−0.00 ± 0.04	.028

Abbreviations: ARCR, arthroscopic rotator cuff repair; CAIO, closed reduction and internal fixation of femur; EABM, excision and biopsy mass; KAM, knee arthroscopy/meniscectomy; THA, total hip arthroplasty; TKA, total knee arthroplasty; K-DRS-R-98, Korean version of the Delirium Rating Scale–Revised–98; MDS-OAβ, Multimer Detection System-Oligomeric Amyloid-β.

^a^Statistical test was done between patients with POD and without POD.

^b^Within the Delirium group, postMDS-OAβ was significantly higher than preMDS-OAβ (paired t-test, *P* = .017).

### Detection of delirium and sample collection

Participants were evaluated for POD once daily for the first three postoperative days by trained research staff; when delirium was suspected in any setting (ward or ICU), the Korean version of the Delirium Rating Scale-Revised-98 (K-DRS-R-98) was administered, and POD diagnosis followed previously published thresholds whereby K-DRS-R-98severity ≥18.5 or K-DRS-R-98total >20.5 were considered diagnostic [23], with daily nursing consultations used to corroborate findings. Blood sampling was performed twice per patient: preoperatively on the day before surgery (with routine blood tests), and postoperatively at first POD identification or, if no POD occurred within postoperative Days 1–3, on the morning of postoperative Day 4.

### The measurement of oligomerization of Aβ in plasma

Plasma Aβ oligomerization was quantified using the MDS-OAβ assay (PeopleBio Inc.), performed by an external laboratory according to the manufacturer’s standard operating procedure, with a single determination per sample. The manufacturer-reported lower limit of quantification is 0.063 ng/ml, with a reliable detection range of 0.313–10 ng/ml [19]. Briefly, frozen plasma was thawed at 37°C for 15 min; synthetic Aβ was added; mixtures were incubated for 48 h at 37°C to promote oligomer formation; incubated samples and serially diluted standards were then dispensed into assay wells and incubated 1 h at room temperature. After addition of enhanced chemiluminescence substrate (100 μL/well; Rockland Immunochemicals, Limerick, PA, USA), relative luminescence units (RLUs) were read on a Victor 3 spectrophotometer. All assays were conducted in a laboratory blinded to clinical data and outcomes.

### Statistical analysis

Continuous variables were summarised as mean ± SD and categorical as n (%). POD vs. no-POD comparisons used two-sample t tests and χ^2^/Fisher’s exact tests (two-sided α = 0.05); Welch’s t test was applied when variances were unequal. Primary contrasts were preoperative MDS-OAβ, postoperative MDS-OAβ and ΔMDS-OAβ (post–pre). To address confounding, all contrasts were repeated in a propensity-score–matched (PSM) cohort centred on APOE ε4 (logistic PS for P[POD = 1|ε4]; 1:1 nearest-neighbour without replacement; calliper 0.5 × SD of the logit; each subject used once). Balance was assessed by standardised mean differences (target <0.10) and Love plots. Associations with K-DRS-R-98severity/total used Spearman’s ρ (matrix α = 0.01). Discrimination was summarised by ROC AUC with DeLong CIs (bootstrap checks). Fixed preoperative cut-points (0.60/0.72/0.85 ng/ml), selected as ROC-derived operating points to reflect pragmatic clinical targets (low-risk, intermediate-risk and high-risk decision points), were applied as-is to report sensitivity, specificity, predictive values and likelihood ratios (95% CIs) without further tuning. For sensitivity, we pooled postoperative data from a prior retrospective cohort (*n* = 104) with the present cohort (*n* = 101) (total *n* = 205) and compared AUCs by DeLong and sensitivities/specificities at fixed cut-points by two-proportion z tests. Exploratory model-based metrics (ΔAUC, category-free NRI/IDI), calibration (intercept/slope) and decision-curve analysis compared a clinical model (age, sex, education, MMSE, APOE ε4) with a clinical+biomarker model; diagnostics included VIFs, logit linearity (restricted cubic splines) and influence checks (studentised residuals, leverage, Cook’s distance). No routine winsorization/exclusion was applied; additional sensitivity runs were reported only if influential points altered conclusions. Unless noted, PSM analyses mirrored the overall analyses (same hypotheses and fixed thresholds).

## Results

### Clinical characteristics and MDS-OAβ in all participants and by POD status

**Figure 1:**
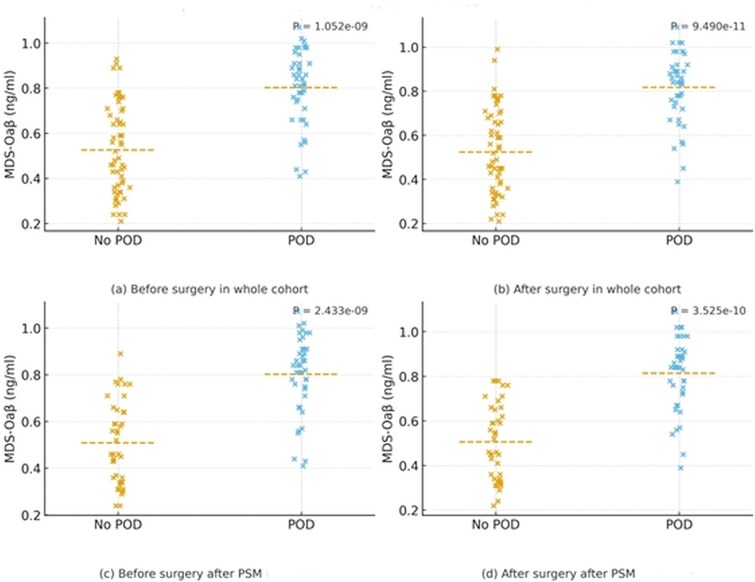
Scatter plots of plasma MDS-OAβ levels before and after surgery in patients with and without postoperative delirium: whole cohort and PSM cohort. Scatter plots show serum MDS-OAβ (ng/ml) levels in patients with and without postoperative delirium (POD) before and after surgery. Dashed horizontal lines indicate group means, and *P*-values were calculated using the independent two-sample t-test (Welch’s correction).

Among 101 patients (POD 44; no-POD 57), baseline age, sex, education, MMSE and APOE ε4 were similar between groups. The surgical case-mix reflected typical older orthopaedic procedures (Table 1). Delirium ratings were higher in POD (K-DRS-R-98severity 24.0 vs. 4.0; total 26.9 vs. 4.2). Pre-op MDS-OAβ was higher in POD (0.80 vs. 0.53 ng/ml, *P* < .001), as was post-op (0.82 vs. 0.52 ng/ml, *P* < .001). The within-subject change was small but different (Δ = 0.01 ± 0.04 vs. 0.00 ± 0.04 ng/ml, *P* = .028). Within POD, post-op exceeded pre-op (paired *P* = .017), with no change in no-POD (Table 1 and Figure 1).

### Multivariable model with clinical covariates and rationale for PSM

In a multivariable model including age, sex, education, MMSE and APOE ε4 allele count, preoperative MDS-OAβ remained independently associated with POD (B = 0.825, SE = 0.169; *P* < .001) (Table 2). APOE ε4 allele count did not reach statistical significance but showed a trend (B = 0.804, SE = 0.485; *P* = .098). Given the biological plausibility of APOE ε4 as a confounder of amyloid-related measures and delirium vulnerability, we centred propensity score matching (PSM) on APOE ε4 allele count to mitigate confounding in subsequent analyses.

**Table 2:** Multivariable logistic regression model for postoperative delirium including preoperative MDS-OAβ and clinical covariates (*n* = 101)

Variables	B (log-odds)	SE(B)	OR = exp(B)	95% CI for OR	*P*-value
Age	−0.046	0.052	0.95	0.86–1.06	.377
Sex	−0.785	0.560	0.46	0.15–1.37	.161
Education	−0.157	0.099	0.86	0.70–1.04	.113
MMSE	−0.061	0.235	0.94	0.59–1.49	.796
Number of ApoE4 alleles	0.804	0.485	2.23	0.86–5.79	.098
Preoperative MDS-OAβ	0.825	0.169	2.28	1.64–3.18	<.001

Values are regression coefficients (B), standard errors [SE(B)], odds ratios (OR) with 95% confidence intervals and *P*-values from multivariable logistic regression. Preoperative MDS-OAβ is scaled per 0.1 ng/ml increase. Clinical covariates include age, sex, education, MMSE and APOE ε4 allele count.

### Clinical characteristics and MDS-OAβ in PSM cohort and by POD status

PSM yielded 82 patients (41 POD; 41 no POD) with balanced covariates (Table 3). This PSM cohort was a matched subset of the original study population (*n* = 101), not an independent cohort. Baseline comparisons were similar between groups (all *P* ≥ .091). Between-group differences in MDS-OAβ persisted after matching: pre-op 0.80 vs. 0.51 ng/ml (*P* < .001) and post-op 0.81 vs. 0.51 ng/ml (*P* < .001). The mean ΔMDS-OAβ was small and did not differ significantly between groups (0.01 ± 0.03 vs. 0.00 ± 0.03 ng/ml; *P* = .096). Within the POD group, post-op values exceeded pre-op (paired t-test *P* = .0129). Distributions are visualised in Figure 1.

**Table 3:** Clinical characteristics and MDS-OAβ value in the PSM cohort, patients with and without postoperative delirium

Variables	Overall, *n* = 82	Delirium		*P*-value^a^
		Yes, *n* = 41	No, *n* = 41	
Age, years	76.0 ± 5.7	76.3 ± 5.7	75.6 ± 5.7	.576
Female gender (%)	35 (42.7%)	16 (39.0%)	19 (46.3%)	.503
Education	8.5 ± 3.3	7.9 ± 2.9	9.0 ± 3.6	.198
MMSE	28.2 ± 1.2	28.0 ± 1.2	28.4 ± 1.1	.091
Number of ApoE4 alleles	0.3 ± 0.6	0.3 ± 0.6	0.3 ± 0.6	1.000
Surgery name				
THA	45	23	22	–
KAM	9	6	3	–
ARCR	9	4	5	–
TKA	9	4	5	–
CAIO	8	3	5	–
EABM	2	1	1	–
K-DRS-R-98severity	14.1 ± 10.3	24.1 ± 3.1	4.1 ± 1.6	.000
K-DRS-R-98total	15.7 ± 11.7	27.0 ± 3.4	4.3 ± 1.5	.000
preMDS-OAβ(ng/ml)	0.66 ± 0.22	0.80 ± 0.17	0.51 ± 0.18	.000
postMDS-OAβ(ng/ml)	0.66 ± 0.22	0.81 ± 0.16^b^	0.51 ± 0.17	.000
delta MDS-OAβ(ng/ml)	0.00 ± 0.03	0.01 ± 0.03	0.00 ± 0.03	.096

Abbreviations: K-DRS-98, Korean version of Delirium Rating Scale-98; MDS-OAβ, Multimer Detection System–Oligomeric Amyloid-β.

^a^Statistical test was performed between patients with POD and those without POD.

^b^Within the Delirium group, postMDS-OAβ was significantly higher than preMDS-OAβ (paired t-test, *P* = .0129).

### Correlations with delirium burden (whole and PSM cohorts)

Spearman correlation matrices showed strong positive associations between MDS-OAβ and delirium burden in both datasets (Appendix 1 in the Supplementary Data). In the whole cohort (*n* = 101), preoperative MDS-OAβ correlated with K-DRS-R-98severity and total, and postoperative MDS-OAβ correlated with severity and total (all *P* < .01). The correlation between pre- and postoperative MDS-OAβ was ρ = 0.983 (*P* < .01). In the PSM cohort (*n* = 82), preoperative MDS-OAβ correlated with severity and total, and postoperative MDS-OAβ correlated with severity and total (all *P* < .01); the pre–post correlation was ρ = 0.987 (*P* < .01).

### Discrimination of preoperative MDS-OAβ and dual-threshold operating points

**Figure 2:**
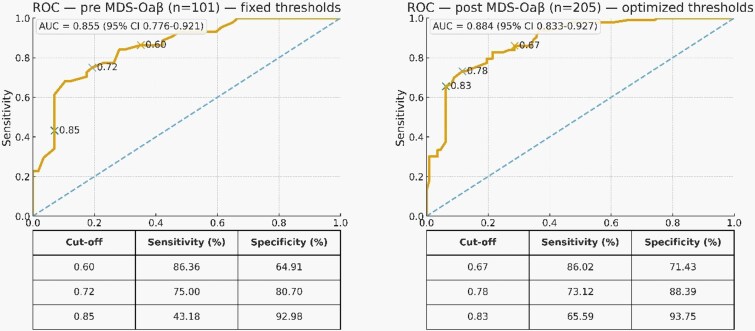
ROC curves for MDS-OAβ predicting postoperative delirium: preoperative (*n* = 101, fixed thresholds) and postoperative pooled (*n* = 205, optimised thresholds). Left: preoperative ROC (*n* = 101) with fixed thresholds 0.60/0.72/0.85. Right: pooled postoperative ROC (*n* = 205) with optimised thresholds 0.67/0.78/0.83. Solid curves = ROC; dashed diagonal = chance. AUC (95% CI) by DeLong (bootstrap-checked). Markers denote thresholds; tables report sensitivity/specificity. Axes: 1–specificity (x) and sensitivity (y).

Preoperative MDS-OAβ showed good discrimination for POD (AUC = 0.855; 95% CI, 0.777–0.919; SE = 0.037). Using fixed clinical thresholds, performance in the preoperative cohort (*n* = 101) was: 0.60 (low-risk threshold)—sensitivity 86.36%, specificity 64.91%; 0.72 (intermediate-risk threshold)—sensitivity 75.00%, specificity 80.70%; 0.85 (high-risk threshold)—sensitivity 43.18%, specificity 92.98% (Figure 2). In a pooled postoperative dataset that combined the prior retrospective cohort (*n* = 104) with the present cohort (*n* = 101) under harmonised definitions (total *n* = 205), discrimination was similar (AUC = 0.884; 95% CI, 0.837–0.925), and the AUC did not differ from the preoperative AUC by DeLong’s test (z = 0.65, *P* = .51). Applying the same fixed thresholds to the pooled postoperative data yielded: 0.60—sensitivity 93.55%, specificity 60.71%; 0.72—sensitivity 82.80%, specificity 78.57%; 0.85—sensitivity 59.14%, specificity 93.75% (Figure 2). An exploratory, dataset-specific optimisation for the postoperative data identified cut-points of 0.67 (low-risk; sensitivity 86.02%, specificity 71.43%), 0.78 (intermediate risk; sensitivity 73.12%, specificity 88.39%) and 0.83 (high-risk; sensitivity 65.59%, specificity 93.75%), broadly aligning with the fixed operating points (Figure 2). Between-dataset comparisons at the same thresholds showed no significant differences at 0.60 and 0.72 for both sensitivity and specificity (all *P* ≥ .165); at 0.85, the pooled postoperative set showed a trend toward higher sensitivity (z = −1.75, *P* = .080) with similar specificity (*P* = .848). Overall, the fixed dual-threshold operating points generalised well across samples and sampling time-points.

## Discussion

We approached this work with a vulnerability-first hypothesis: if plasma oligomeric Aβ indexes a relatively stable substrate of neural risk, pre- and early postoperative values should be tightly coupled and between-group differences chiefly evident at baseline. This expectation drew on prior strong pre/post concordance with small deltas [20], broader links between POD and Alzheimer’s biology, and the practicality of a single preoperative blood test. To contextualise the event rate, POD occurred in 44/101 patients, which may be higher than in some reports and could reflect an older orthopaedic inpatient case-mix and active daily ascertainment on postoperative Days 1–3. Across both the overall and APOE ε4-matched cohorts, preoperative MDS-OAβ discriminated POD and tracked delirium burden, indicating a largely trait-like signal. Superimposed on that substrate, we observed only a small within-patient postoperative rise in the delirium group, with no change in those without delirium; between-group differences in ΔMDS-OAβ were modest and were not statistically significant after APOE ε4-centred matching. Thus, vulnerability remains primary, while perioperative physiology unmasks a modest state change selectively in susceptible patients. These findings are consistent with prior perioperative MDS-OAβ studies showing higher preoperative values in patients who develop POD and limited perioperative change, supporting a vulnerability-first framework [20]. Although a small within-patient rise was observed in the POD group, the effect size was modest; the between-group ΔMDS-OAβ difference was significant in the overall cohort (*P* = .028) but was no longer significant after APOE ε4-centred propensity matching (*P* > .05), indicating that baseline vulnerability is the dominant signal. Prospective and quantitative syntheses show that lower preoperative CSF Aβ42 predicts incident POD and early postoperative cognitive decline, consistent with latent neurodegenerative vulnerability under surgical stress [4, 5]. By contrast, small-scale PET studies of cortical amyloidosis show inconsistent associations with POD severity, and recent plasma work using monomeric Aβ42/40 was negative [6–8]. Together with our findings, this pattern supports the view that oligomeric biology lies closer to clinically active mechanisms in older surgical populations [24].

The MDS-OAβ quantifies plasma oligomerization tendency and has shown associations with amyloid burden across the AD continuum [12, 13], although stage-dependent (potentially non-monotonic) patterns have been reported [13]; ApoE-ε4–related differences in oligomer handling bolster plausibility in elders [14]. We acknowledge that plasma p-tau217 is among the most widely validated blood biomarkers for AD pathology, but it was not measured in this cohort; head-to-head evaluation with MDS-OAβ and broader AT(N) panels for POD prediction is therefore a priority for future studies [25]. However, POD is a multifactorial syndrome in which baseline vulnerability interacts with perioperative triggers; therefore, greater AD-specificity does not necessarily translate into superior POD prediction. Accordingly, we position MDS-OAβ as a complementary oligomer-axis vulnerability marker—biologically plausible for synaptotoxicity/network-level fragility—intended to add actionable information alongside clinical risk tools rather than replace established AD biomarkers. Direct head-to-head comparisons with p-tau217 and broader AT(N) panels were not available in this cohort and are a priority for future external validation.

In Korean practice, MDS-OAβ is used adjunctively when AD is suspected, with a commonly referenced cut-off near 0.78 ng/ml [15–18] although memory-clinic thresholds need not map directly to perioperative decisions, they offer a familiar anchor as action thresholds are calibrated for risk management. Our data align with earlier programmatic work. In a retrospective orthopaedic-predominant cohort, higher postoperative MDS-OAβ tracked POD incidence and delirium severity [19]. In a prospective pilot with standardised pre-/postoperative sampling and blinded adjudication, pre- and postoperative values were tightly coupled and the pre-to-post change was small [20]. Here, we again observe strong coupling (ρ ≈ 0.98) and add that a small uptick emerges only in POD. Overall, the pattern supports a stress–vulnerability model: a stable oligomeric predisposition dominates risk, while perioperative triggers reveal a selective, modest perturbation.

Two non-exclusive mechanisms remain plausible. In a vulnerability model, elevated oligomerization reflects impaired proteostasis and synaptic fragility along amyloidogenic pathways, with ε4-related differences in oligomer kinetics/clearance shaping risk [14]. In a trigger-plus-substrate model, perioperative exposures transiently potentiate oligomerization in predisposed individuals; preclinical studies indicate certain inhaled anaesthetics can enhance Aβ oligomerization and cytotoxicity [26, 27]. The small Δ confined to POD, with near-preserved pre/post rank order, is consistent with a baseline substrate governing overall risk and acute physiology revealing a subtle, state-dependent amplification rather than a universal surgical artefact.

For clinical translation, a dual-threshold framework is pragmatic. We selected three ROC-derived preoperative operating points to reflect pragmatic clinical targets: 0.60 ng/ml emphasising sensitivity/NPV (low-risk), 0.72 ng/ml as an intermediate risk and 0.85 ng/ml emphasising specificity/PPV (high-risk). Because the high-risk threshold prioritises specificity, its sensitivity is lower and a proportion of POD cases will not be captured; thus, it is intended to prioritise targeted prevention resources rather than serve as a stand-alone screening threshold. We emphasise that these thresholds are intended for risk stratification rather than definitive diagnostic thresholds of delirium. A value below the low threshold may support de-escalation to standard prevention bundles, but it does not exclude POD, given delirium’s multifactorial triggers and biomarker overlap. Conversely, the high threshold prioritises specificity to identify a high-risk group for targeted prevention, acknowledging that sensitivity is lower at this operating point and some POD cases will be missed. Preoperative MDS-OAβ achieved good discrimination (AUC 0.855; 95% CI, 0.777–0.919) and interpretable performance at these fixed cut-points. In a pooled postoperative sensitivity analysis combining a prior retrospective cohort (*n* = 104) with the present cohort (*n* = 101) under harmonised definitions (*n* = 205), discrimination was similar (AUC 0.884; 95% CI, 0.837–0.925), with no significant AUC difference vs. pre-op by DeLong. Applying the same thresholds to pooled postoperative data yielded comparable sensitivity/specificity, suggesting operating characteristics generalise across perioperative sampling windows despite a small Δ occurring selectively in POD. Operationally, values <0.60 ng/ml could support de-escalation to standard bundles; ≥0.85 ng/ml could trigger targeted prevention (enhanced reorientation/mobilisation, sleep protection, sensory optimisation, multimodal opioid-sparing analgesia, early family engagement, consultation pathways). The balanced point (~0.72 ng/ml) lies near the 0.78 ng/ml memory-clinic benchmark [15–18], lending face validity even as perioperative prevalence and action thresholds differ. Where postoperative blood is feasible, modest gains in rule-in sensitivity may be observed while preserving specificity; nevertheless, thresholds should be prespecified, applied as-is and prospectively monitored for calibration. However, given the overlap in preoperative values, MDS-OAβ is best interpreted as an adjunct marker for perioperative risk stratification rather than a stand-alone discriminator.

Methodologically, we centred propensity-score matching on APOE ε4 to address a biologically plausible confounder. ε4 showed a statistical trend in the clinical-only model and has links to oligomer dynamics and delirium vulnerability [28, 29], providing a principled anchor for matching. After ε4-centred PSM, the between-group MDS-OAβ difference remained, supporting consistency after balancing a key confounder. However, because PSM restricts the analysis to matchable patients, findings from the matched subset should be interpreted as a sensitivity analysis rather than as an estimate of real-world predictive performance. Several features enhance credibility and translation: standardised, blinded delirium adjudication (K-DRS-98); discrimination summarised both by AUC and by prespecified cut-points with likelihood ratios and predictive values; convergent results in whole and matched cohorts; and thresholds positioned relative to an external benchmark familiar in Korean practice (0.78 ng/ml).

Limitations qualify interpretation. This was a single-centre, orthopaedic-predominant study with moderate size; generalizability to other procedures, anaesthetic techniques and perioperative pathways requires multicentre validation [30, 31], and we did not apply a delirium-specific standardised assessment immediately preoperatively, so some cases labelled as POD may have had evolving preoperative delirium [32]. ε4-focused matching improves internal validity for a key pathway but does not exclude residual confounding [31]. We did not model key perioperative triggers—particularly systemic inflammation, as well as anaesthetic/analgesic exposure and sleep/circadian disruption—which limits mechanistic inference about delirium episodes and supports interpreting MDS-OAβ primarily as a preoperative vulnerability marker for risk stratification. Despite daily ratings, delirium’s fluctuating course risks under-ascertainment. Finally, we used three fixed operating points without re-fitting; although pooled postoperative analysis suggests stability across sampling windows, pre-analytic harmonisation and lot-to-lot quality assurance remain essential for transportability. These caveats define next steps. Multicentre validation across surgical domains should prospectively test transportability of the ROC-derived decision thresholds and assess calibration drift. Compact clinical models—limited to a small set of perioperative variables—should be combined with MDS-OAβ to quantify incremental discrimination and net benefit (e.g. decision-curve analysis). Time-series designs with repeated perioperative sampling could clarify how ΔMDS-OAβ and rate of change relate to delirium onset, peak and resolution. Mechanistic studies integrating APOE genotype, inflammatory profiles, blood–brain barrier permeability, sleep/circadian physiology and anaesthetic exposure may explain why Δ emerges only in POD and identify modifiable levers. Finally, implementation trials should test whether threshold-guided prevention reduces POD incidence/duration, improves functional outcomes and lowers costs in real-world perioperative care.

## Conclusions

Preoperative plasma MDS-OAβ functions as a scalable, clinician-interpretable POD risk marker with prespecified dual thresholds. The selective postoperative rise confined to POD, together with near-identity of pre/post rank order, supports a stress–vulnerability interaction rather than a purely static trait. With good discrimination, familiar operating points and preliminary cross-window stability, the approach supports pragmatic risk stratification today while motivating prospective, multicentre work to confirm transportability, refine calibration and determine whether threshold-guided care improves outcomes for older surgical patients.

## Supplementary Material

aa-25-3403-File004_afag106

## Research Data Transparency and Availability

Anonymised data from this study are available from the corresponding author upon reasonable request for academic purposes.
