# Supplementary material for: Dual-threshold plasma Aβ oligomerization for postoperative delirium risk stratification
Source: Age Ageing. 2026 Apr 22;55(4):afag106. doi: 10.1093/ageing/afag106 (PMC13100654; doi:10.1093/ageing/afag106)
Supplement: aa-25-3403-File004_afag106 [file aa-25-3403-file004_afag106.docx]

**Appendix 1.** **Spearman Correlation Coefficient Matrices Among K-DRS and Serum MDS-OAβ (pre- and post-operative) in Study Subjects (Whole Cohort and PSM Cohort).**

| Variables | preMDS-OAβ(ng/ml) | postMDS-OAβ(ng/ml) | K-DRS-R-98severity | K-DRS-R-98total |
| --- | --- | --- | --- | --- |
|  |  |  |  |  |
| In Whole cohort(n=101) |  |  |  |  |
| preMDS-OAβ(ng/ml) | 1.0 | 0.983^*^ | 0.733^*^ | 0.739^*^ |
| postMDS-OAβ(ng/ml) |  | 1.0 | 0.743^*^ | 0.754^*^ |
| K-DRS-R-98severity |  |  | 1.0 | 0.986^*^ |
| K-DRS-R-98total |  |  |  | 1.0 |
|  |  |  |  |  |
| In PSM cohort(n=82) |  |  |  |  |
| preMDS-OAβ (ng/ml) | 1.000 | 0.987^*^ | 0.769^*^ | 0.781^*^ |
| postMDS-OAβ (ng/ml) |  | 1.000 | 0.788^*^ | 0.805^*^ |
| K-DRS-R-98severity |  |  | 1.000 | 0.984^*^ |
| K-DRS-R-98total |  |  |  | 1.000 |

K-DRS-R-98; Korean version of the Delirium Rating Scale–Revised–98, MDS-OAβ: Multimer Detection System-Oligomeric Amyloid-β

^*^ P <0.01
